# Recovering of Dizziness of a Patient with Sinusitis after Root Canal Therapy for Upper Second Molar

**DOI:** 10.1155/2019/8394147

**Published:** 2019-12-16

**Authors:** Ibrahem Amro, Lyana Shawar, Raed abu Hantash, Basem Abuquba, Akram Amro

**Affiliations:** ^1^Lebanon Dental Clinic, State of Palestine; ^2^Al-Quds University, State of Palestine

## Abstract

This case report illustrates the recovery of dizziness of a young healthy patient after root canal therapy of upper second molar. The patient developed dizziness and unbalanced walking four months ago. After cardiac, ENT, neurological, physiotherapy and medical investigations, his entire checkup showed no abnormalities. The patient visited a dental clinic for a routine checkup; after dental clinical and radiographical examination, a chronic abscess infection in an upper second molar region close to the sinus was diagnosed. Root canal therapy was performed that resulted in a disappearance of the dizziness and full recovery was achieved. *Conclusion.* Infected upper teeth with periapical lesion are associated with dizziness as a complication of odontogenic-related sinusitis. Dental and medical cooperation contributes to a better management diagnosis of the dizziness.

## 1. Introduction

Dizziness is the sensation of disturbed or impaired spatial orientation without a false or distorted sense of motion [1]; there are many conditions and diseases may cause dizziness. One of the most common diagnoses was sinusitis in addition to labyrinthitis, otitis media, benign positional vertigo, and unspecified presyncope that were among the most common causes of dizziness [2].

Sinusitis is one of the most common respiratory infections as a result of pathogenic bacteria through the oral cavity or nasal ostium [3]. The signs and symptoms of sinusitis may include per orbital edema, headache, facial pain, tooth pain, fever, earache, a sore throat, cough, nasal congestion, dizziness, nasal obstruction, and foul breath [4].

One of the common types of sinusitis is the OS which may occur when the Schneiderian membrane is disrupted by many conditions [5] such as infections which originate from maxillary teeth, maxillary dental trauma odontogenic pathology of maxillary bone, or iatrogenic causes like dental implant, extractions, and maxillary osteotomies in orthognathic surgery [3].

Lee and Lee (2010) investigated the clinical features and treatments of (OS) and found that dental infection was a major predisposing factor of OS.

Brook (2016) reviewed the different management strategies of sinusitis of odontogenic origin; he pointed out that both surgical and dental treatments of the odontogenic pathological conditions combined with medical therapy are indicated.

Root canal therapy is a debridement procedure that is based on the removal of the irritants (bacteria) and inactivation of endotoxins and other toxic products from the canal and periapical areas to get a successful treatment [6], [7]. Root canal treatment depended on chemical and mechanical ways [6]. Mechanical way is based on using manual and rotary files. The rotary system has many benefits; it reduces the intracanal bacteria [8] and increases the quality of root fillings [9].

## 2. Case Presentation

This case study indicates the effectiveness of root canal therapy to treat OS and disappearance of symptoms. Patient consent was obtained, and then history was taken. A 26-year-old male who is medically fit had suffered from dizziness in walking and standing. He had a headache while eating and tilting his head forward.

The patient consulted internal, cardiac, and neurological and ENT specialists to treat the dizziness; all medical investigations were negative and all given conservative management failed to improve his dizziness.

**Figure 1:**
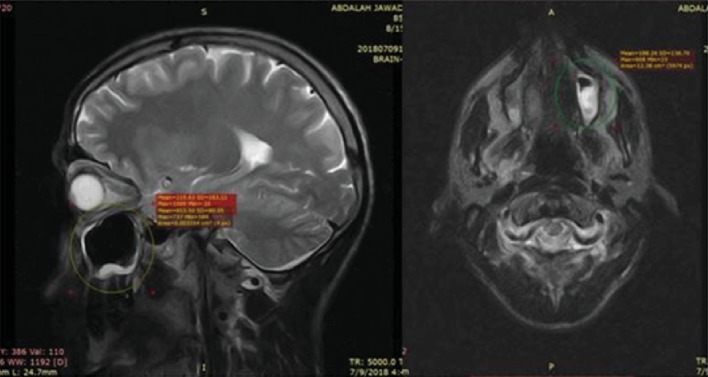
MRI sagittal and axial views show thickness of mucosal layer of maxillary sinus.

After a further audiologist examination, videonystagmography study showed normal study with no signs of peripheral dizziness cause in origin. The static position test at different head positions with and without eye closure showed no significant spontaneous or any positional nystagmus. Cervical spine MRI was normal and brain MRI showed chronic sinusitis (Figure 1).

According to the MRI findings, the patient was treated with many types of antibiotic which were azithromycin 500 mg three times daily/one course and then amoxicillin with clavulanic acid 500 mg three times daily/one course. The physician changed the antibiotic to ciprofloxacin (500 mg twice daily).

**Figure 2:**
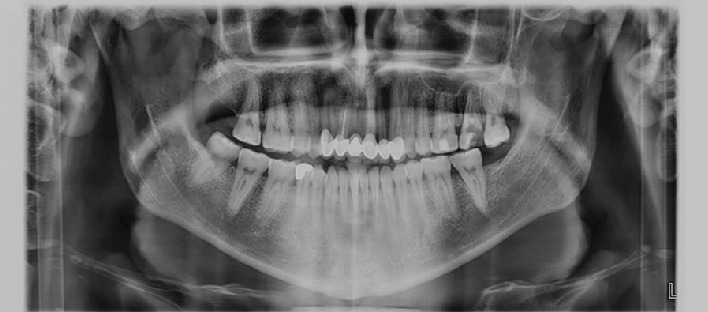
Orthopantomogram before treatment showed the upper left second molar opened with periapical radiolucency.

**Figure 3:**
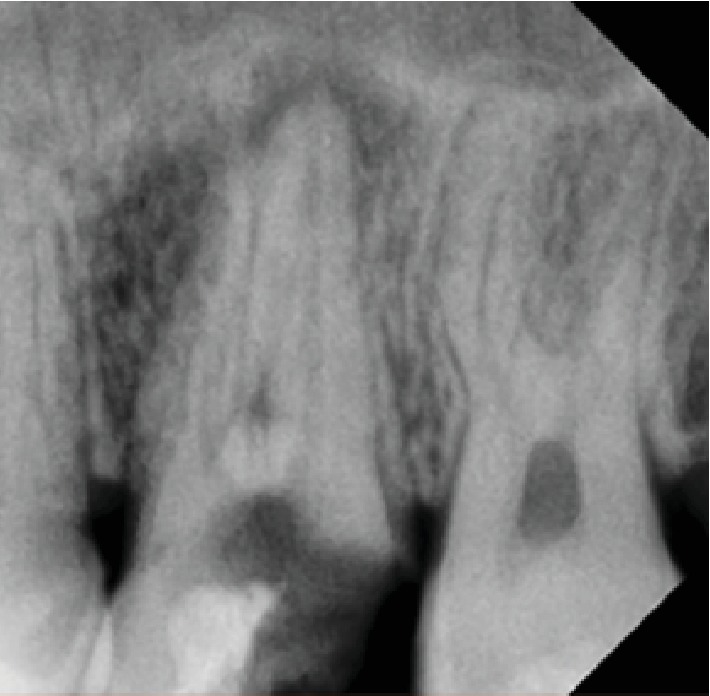
Periapical X-ray before treatment.

**Figure 4:**
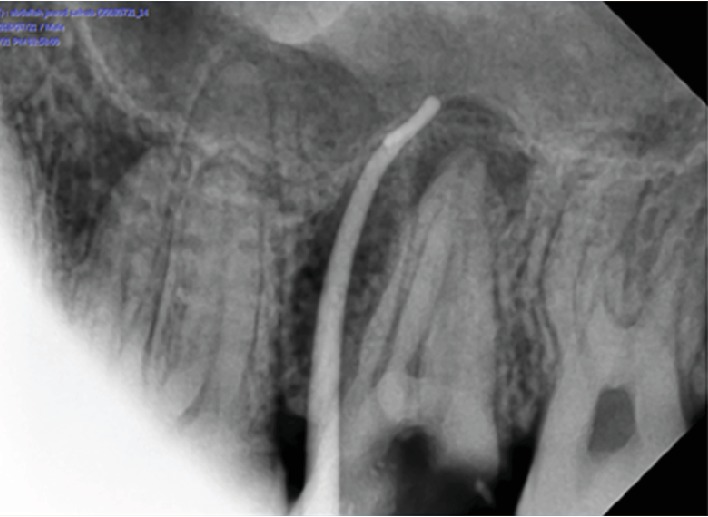
Periapical X-ray shows gutta-percha inserted through sinus.

**Figure 5:**
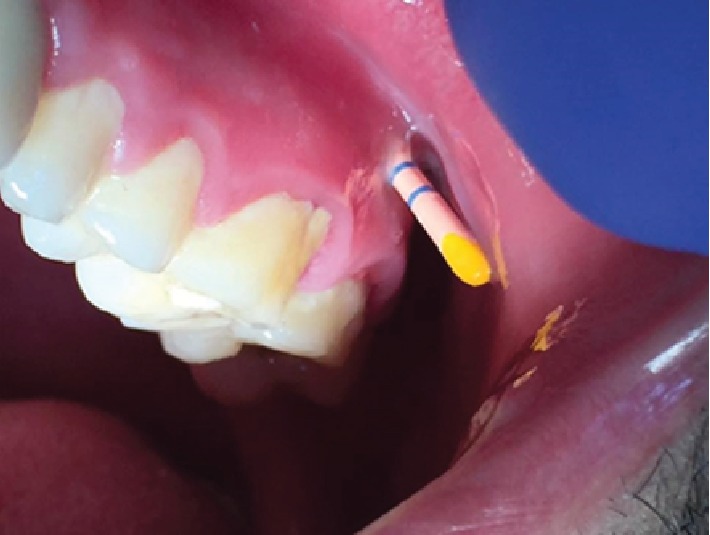
Gutta-percha inserted through sinus.

After this history of none responsive conservative medical management, the patient came for the dental examination and baseline assessment and examination was performed; dental examination showed no swelling or inflammation to be detected; pain was reported as 7 out of 10. Intraoral examination showed sinus tract with pus mobility grade 2. Radiograph examination in orthopantomogram and periapical (Figures 2 and 3) showed radiolucency around upper 6 and 7 and arrived at the border of the maxillary sinus using rubber dam for isolation starting with using a cold and percussion test to determine the vitality of pulp, upper 6 positive on cold but there is no pain on percussion and upper 7 pain on percussion but negative on cold. Our initial diagnosis was upper left second molar previously initiated therapy with chronic apical abscess with sinus tract; the upper left first molar was diagnosed with irreversible pulpitis; normal apical tissues used gutta-percha to determine the caused tooth of sinus track (Figures 4 and 5). According to X-ray, signs, and a gutta test, the upper left second molar was the caused tooth; a management plan was based on root canal therapy to preserve tooth and reduce the sign and symptoms of infection. Preoperative periapical X-ray was taken to determine the initial length of canals. There was no need for anesthesia as it is a nonvital tooth; as mentioned before, the tooth was previously initially treated so the canal orifice was opened, using low-speed turbine to remove the soft dentine and get a good vision. K file numbers 10, 15, and 20 were used to open canals, and normal saline was used as an initial irrigation to get rid of pus.

**Figure 6:**
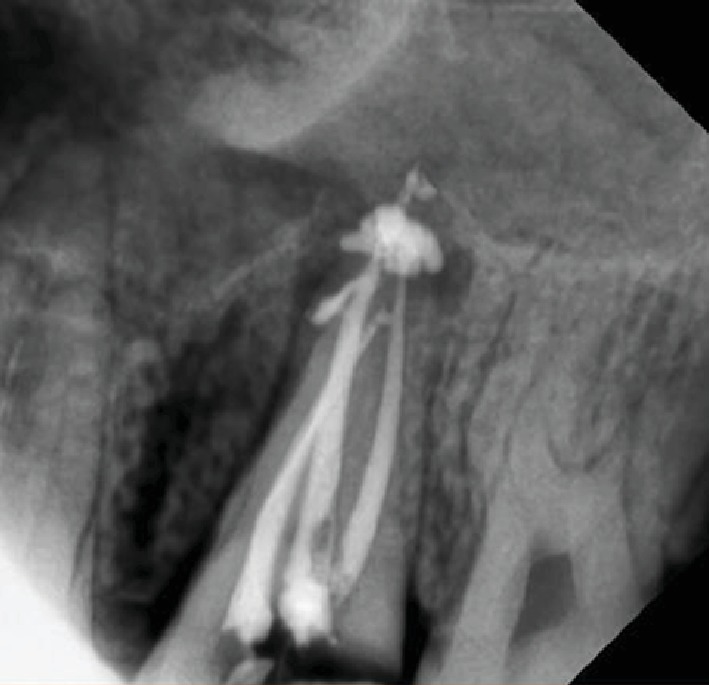
Upper left first molar with calcium hydroxide shows the tooth with direct contact with sinus.

After ensuring that all canals were opened in working length, a rotary system crown-down technique was used to shape canals starting with an initial file in 15 mm sodium hypochlorite 5% that was used for irrigation and hydrogen peroxide, saline between them; protaper rotary system finishing file (F1) was used to finish shaping in full working length of 21 mm. Irrigation with sodium hypochlorite was done, and then normal saline was used for final irrigation, canal was dried using F1 paper point, and calcium hydroxide with iodoform as an internal canal medication (Figure 6).

After one week, the patient showed less dizziness on walking, no pain was reported while eating, and periapical X-ray showed reduce radiolucency CBCT after obturation.

**Figure 7:**
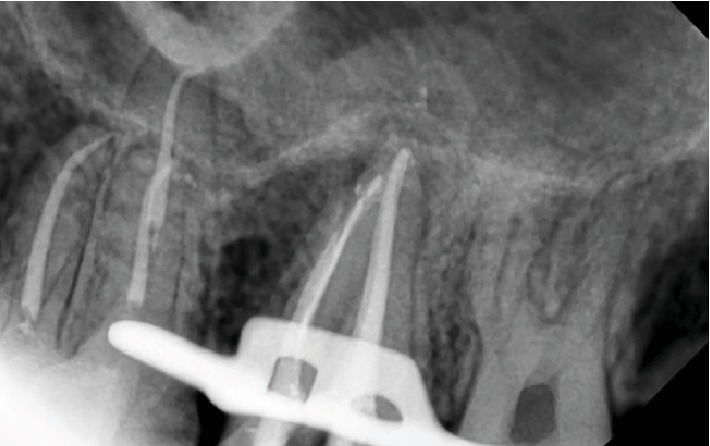
Periapical due to upper 7 obturation.

After 2 weeks further, the patient came to our clinic with severe pain on cold and hot; the upper left first molar was assessed and diagnosed with deep caries. Symptomatic irreversible pulpitis root canal treatment was completed in one visit. After 2 weeks further, using rubber dam isolation treatment started to speed up round bur to remove temporary filling. F1 rotary file had been used to remove all formed calcium hydroxide canals. The process involved irrigation with normal saline, then soaking the canals for around 10 min with sodium hypochlorite, activation (with water pick) for 30 seconds per canal, irrigation with normal saline, and using paper point (F1) to dry the canals. Complete obturation was performed by using F1 Gutta percha and hot Gutta system with endomethazone and eugenol as a sealer. Glass ionomer was used to close the pulp chamber then completing the tooth filling using composite (Figure 7).

**Figure 8:**
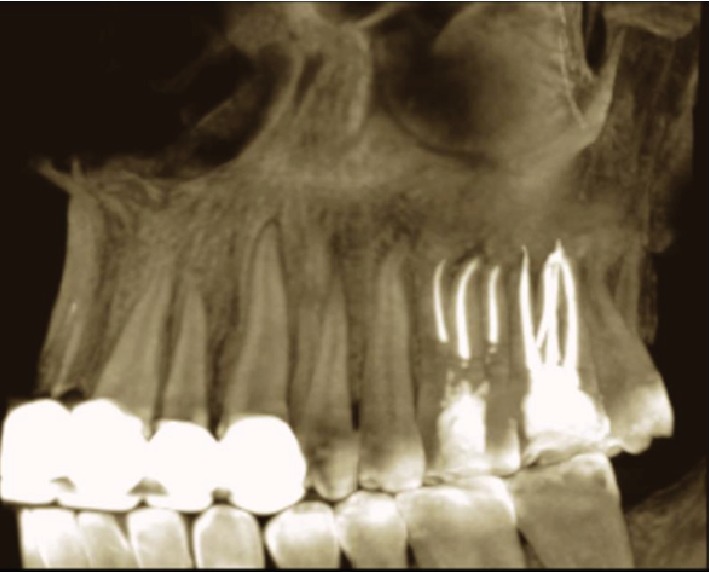
CBCT.

**Figure 9:**
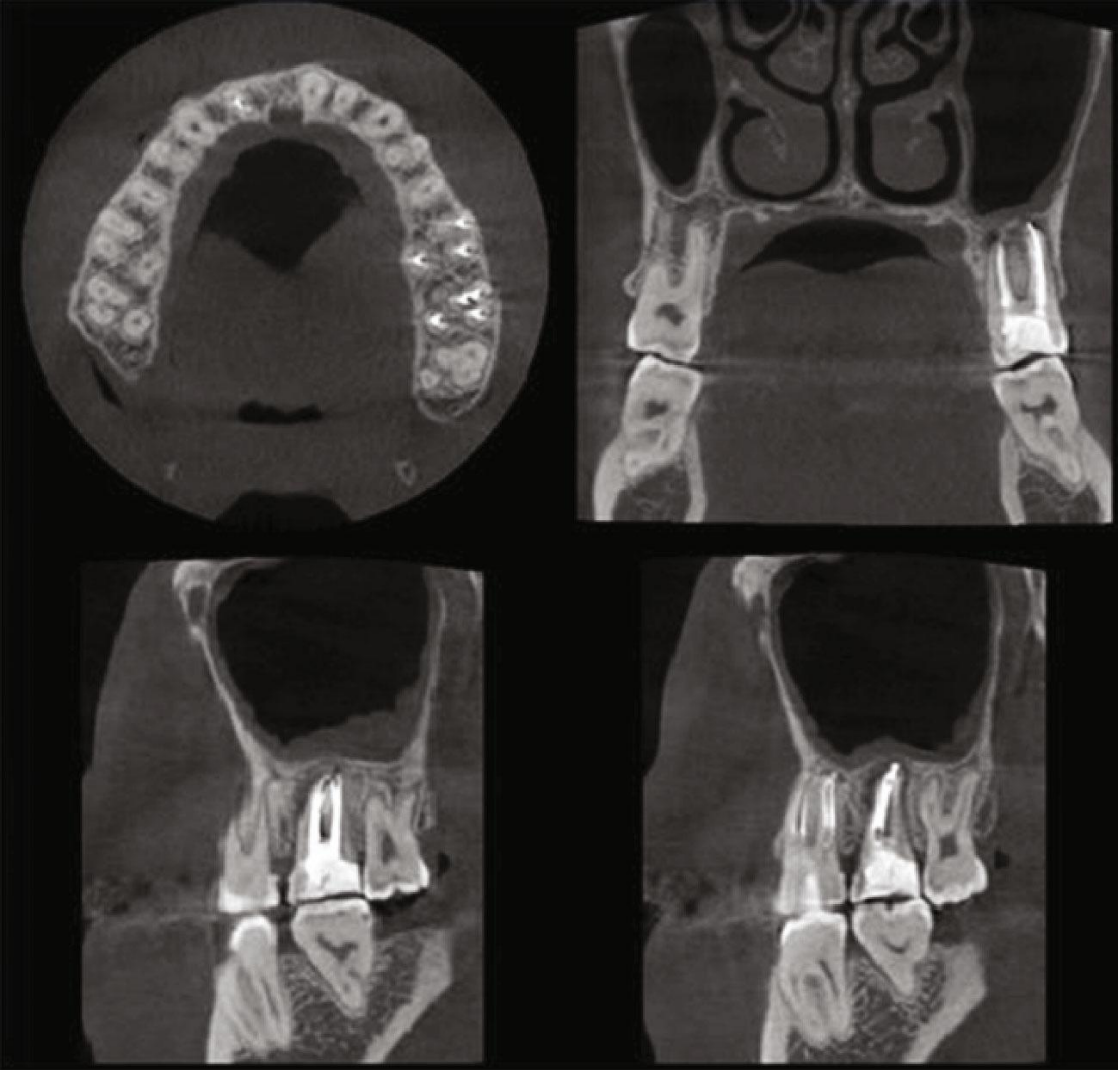
CBCT after obturation. Axial, coronal, and sagittal views.

CBCT after obturation (Figures 8 and 9).

### 2.1. Follow-Up

**Figure 10:**
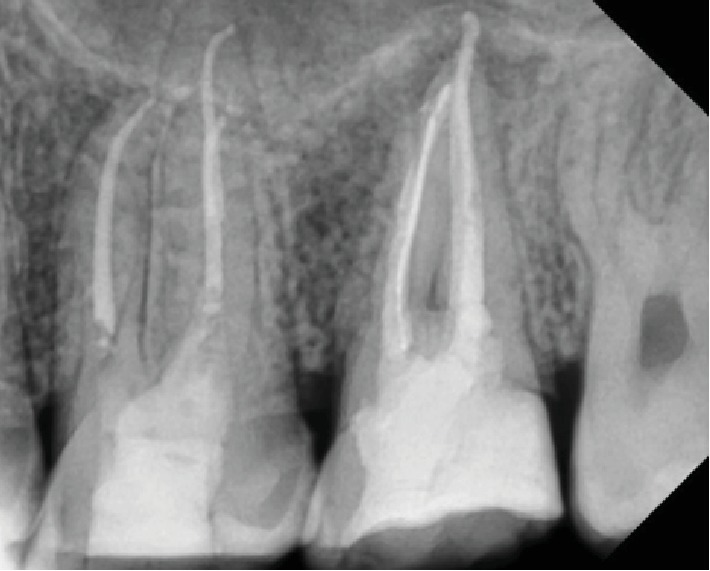
Periapical X-ray after 2 weeks of obturation.

Two months after the intervention, the patient reported a complete recovery of dizziness and sinusitis; pain was reported as 0 m which reflects a primary recovery of OS; a follow-up after 2 weeks periapical X-ray showed decrease of radiolucency (Figure 10).

**Figure 11:**
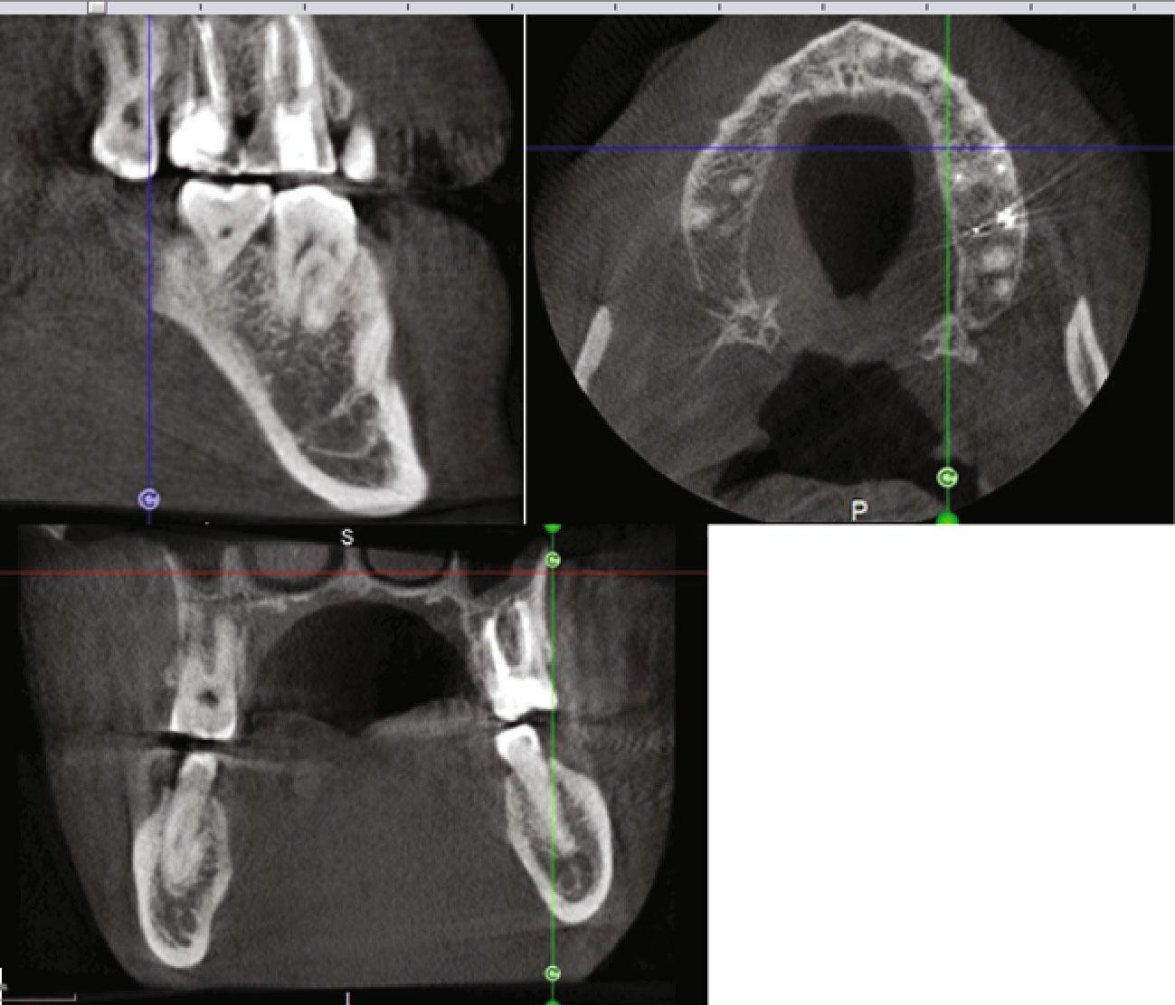
CBCT after 10 months of obturation.

CBCT after 10 months shows disappearance of radiolucency around the apex (Figure 11).

## 3. Discussion

The association between dizziness and sinusitis was not surprising as it was well documented in literature. The auditory tube is a narrow osteocartilaginous channel connecting the nasal cavity with the tympanic cavity [10]. It is a responsibility for pressure equilibrium and ventilation of the middle ear, so according to the anatomical and functional connections, auditory tubes are affected in upper respiratory trunk infection as sinusitis that lead to middle ear and unbalance disorder [11].

OS infections are considered to be predisposing factors for sinusitis, because of the anatomical relation. The antral mucosa has been weakened and broken down and the Schneiderian membrane is perforated [4] by chronic dental infection/inflammation as a reason to bacterial colonization [12].

In the maxillary teeth, the second molar roots are the closest to the maxillary sinus floor, followed by the roots of the first molar, second premolar, and first premolar. These short distances between roots and maxillary sinus explain the easy extension of an infectious process from these teeth to the maxillary sinus [4].

The OS is a polymicrobial infection, aerobic-anaerobic bacteria, with anaerobes outnumbering the aerobes [3]. There are two types of bacteria which are most commonly found in OS, which are *Staphylococcus Aureus* and *Gram-negative bacilli* [3, 13, 14] As was mentioned in patient's history, treatment was based on eliminating this opportunist bacteria.

This justifies why antibiotic treatment without dental treatment failed to eliminate the causing bacteria which lead to treatment failure.

Sinusitis is a common etiology of dizziness, but sinusitis of odontogenic cause associated with dizziness had not been discussed in literature according to the knowledge of the authors, which is the main finding of this article.

This case report showed that there is a correlation between dental infection and sinusitis, which makes the sinus vulnerable for a major source of infection, especially with bad oral hygiene patient. Many patients with maxillary sinusitis were treated symptomatically without treating the underlying causes.

In this case, dizziness was recovered, after the sinusitis had improved, with no major intervention other than root canal therapy, which shows the importance of the proper diagnosis and management of the primary cause, rather than treating temporarily the signs of a complication.

The implication of this finding may be that patients that come with dizziness and the evidence of sinusitis, periapical lesions should be investigated. In this case, it was showed that an open source of contamination will lead to the failure of any pharmacological management of local sinusitis on the long run, which makes this finding an essential element in future history taking from patients with sinusitis-related dizziness.

One of the limitations of this study was that it did not take into consideration the types of bacteria that caused OS to find out which types of bacteria could help in the treatment and could also contribute to a better understanding of this medical interaction. The authors of this study recommend further research in this field, trying to investigate this medical complication with related bacteria or normal flora of oral cavity.

The finding in this case underlines the necessity of management of the infected maxillary teeth not just to preserve the tooth health but also to prevent further complication that sinusitis is only one example of them, with all its complication starting from with dizziness and ending up of tumor [15].

## 4. Conclusion

Failure to diagnose sinusitis of dental origin will lead to further complications including dizziness. Elimination of bacterial sources is essential for preventing recurrence of sinusitis and its chronicity. Referral of sinusitis patient to the dentist may augment treatment and accelerate healing in OS-related dizziness.

Recommendations of this study include the evaluation of any dizzy patient must include dental history and examination, to exclude any causes of dental origin.
